# Large Animal Models of Tricuspid Regurgitation: Relevance, Limitations, and Future Directions

**DOI:** 10.1007/s12265-026-10798-0

**Published:** 2026-06-08

**Authors:** Talia Sukienik, Kosuke Nakamae, Chihiro Miyagi, Grisha Yelisetty, Syed Faizullah Hussaini, Tatsuya Watanabe, Junya Matsuda, Satoshi Yuhara, Daisuke Onohara

**Affiliations:** 1 Center for Regenerative Medicine, Research Institute at Nationwide Children’s Hospital, Columbus, OH USA; 2 Department of Biomedical Engineering, The Ohio State University College of Engineering, Columbus, OH USA; 3 Department of Pediatrics, The Ohio State University College of Medicine, Columbus, OH USA; 4 Center for Regenerative Medicine, Department of Pediatrics, Research Institute at Nationwide Children’s Hospital, Ohio State University College of Medicine, 575 Children’s Crossroad, RB4, Columbus, OH 43215 USA

**Keywords:** Tricuspid regurgitation, Functional tricuspid regurgitation, Large animal models, Pulmonary hypertension, Right heart failure

## Abstract

Despite growing interest in tricuspid regurgitation (TR), evidence remains insufficient to define the optimal timing of intervention and therapeutic strategies, highlighting the need for robust experimental models. Existing large animal models of TR use diverse approaches and can be grouped into two categories: direct valvular interventions targeting the tricuspid leaflets, annulus, or chordae, and indirect induction through mechanisms such as increased right ventricular loading, pulmonary hypertension, or pacing-induced tachycardia. Although direct valvular interventions can reproducibly induce TR and are well suited for modeling primary TR, clinically prevalent secondary TR is driven by annular dilation, leaflet tethering, and right-sided chamber remodeling rather than intrinsic valvular injury. This gap represents a major limitation in the field. In this review, we compare large animal models of TR and outline key considerations to guide model selection, with a focus on approaches that more faithfully reproduce the pathophysiology of TR for mechanistic and translational research.

**Graphical Abstract:**

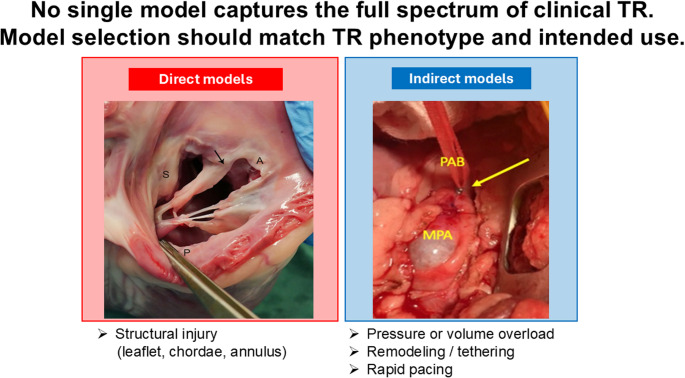

## Introduction

Tricuspid regurgitation (TR) affects more than 70 million individuals worldwide and is an independent predictor of mortality and morbidity when left untreated [1, 2]. Although trivial TR is often observed in healthy individuals, clinically significant TR is associated with progressive right-sided remodeling and adverse clinical outcomes [3, 4]. Functional TR (FTR) is the predominant form, accounting for approximately 70–90% of cases [1, 2], and frequently coexists with pulmonary hypertension (PH) [5]. Importantly, FTR is a heterogeneous entity that includes atrial and ventricular phenotypes, in which the relative contributions of annular dilation and leaflet tethering vary with right atrial (RA) and right ventricular (RV) remodeling patterns.

Despite this substantial clinical burden of TR, current AHA/ACC guidelines state that the optimal timing of intervention for asymptomatic TR is unclear [6]. Surgical repair strategies and transcatheter therapies have expanded [7, 8], yet evidence to guide patient selection and procedural timing remains limited. Many aspects of tricuspid valve (TV) anatomy and function, including annular dynamics and subvalvular geometry, remain incompletely understood. Commonly used experimental approaches induce TR through direct leaflet or subvalvular injury, which are well suited to modeling primary TR but may not capture remodeling-driven secondary (functional) TR, where annular dilation and leaflet tethering contribute to leaflet malcoaptation.

 For these reasons, reliable and reproducible experimental models that accurately replicate the key mechanisms underlying TR are essential for advancing pathophysiological research and preclinical development of procedures and devices. Although small animal models are cost-effective and valuable for investigating molecular mechanisms and early disease pathways, their smaller heart size and anatomical and hemodynamic differences from humans limit their applicability to translational TR research [9]. In contrast, large animal models provide cardiac dimensions, hemodynamic conditions, and procedural access that more closely approximate the clinical setting, making them a more suitable platform for evaluating procedural feasibility, device performance, and safety. Therefore, the development of large animal models is particularly important for translational research and preclinical device development.

By clarifying the strengths and limitations of available large animal models, this review aims to help investigators select experimental platforms that better match the clinical phenotype of interest and support the preclinical evaluation of emerging surgical and transcatheter therapies for TR.

## Anatomical Considerations of the TV

The normal TV complex consists of a fibrous annulus, chordae tendineae, papillary muscles, and three principal leaflets: anterior, septal, and posterior [10]. The number of TV leaflets ranges from three to as many as seven [11]. The anterior leaflet is the largest and most robust, with a quadrangular shape; the posterior leaflet is generally triangular; and the septal leaflet is the most fixed and has a semicircular shape. The septal leaflet is characterized by scalloped indentations with chordae tendineae often arising directly from the interventricular septum [12].

These anatomical features also underlie the distinct mechanisms of TR. Primary TR reflects intrinsic abnormalities of the TV apparatus and is commonly modeled through direct intervention on these components [2, 6, 8, 10, 13]. In contrast, FTR is driven by tricuspid annular dilation and leaflet tethering that develop as part of right-sided remodeling under pressure or volume overload [6, 14, 15]. Accordingly, the induction method used in TR animal models should be matched to the intended disease phenotype.

### Direct Structural Intervention Models (Primary TR)

Direct structural intervention models offer procedural control over TR onset and severity and can be used as acute hemodynamic preparations or as survival models to study chronic right-heart remodeling, depending on the method and follow-up duration (Table 1).

**Table 1:** Animal models of primary tricuspid regurgitation based on direct structural intervention models

Author, year [ref#]	Animal species	Body weight (kg)	Animal number	Methods	Duration		Survival rate [%]				TR
*Leaflet injury or malcoaptation models*											
Xie et al. 2016 [15]	Beagle dogs	14.9 ± 0.7	Intervention: *n* = 11, control: *n* = 3	• Right thoracotomy approach • Resecting one or two tricuspid valve leaflets	Up to 3 years		100				Severe TR: *n* = 11
Yan et al. 2021 [16]	Swine	71.68 ± 7.70	11	• Transvenous approach • Ventricular-side leaflet injury	1 month (*n* = 4); 3 months (*n* = 7)		100				Moderate TR: *n* = 1, Severe TR: *n* = 10
Heerdt et al. 2001 [17]	Mongrel dogs	22–24	8	• Median sternotomy approach • Immobilization of one or two leaflets by applying a traction suture	-		100				Moderate TR: Immobilization of one leaflet, Severe TR: Immobilization of two leaflets
*Subvalvular disruption models*											
Hoppe et al. 2007 [18]	Female ovine	50–75	7	• Transvenous approach • avulsing papillary muscles using a transvenous wire-loop technique	-			100		Not assessed	
Miller et al. 1986 [19]	juvenile swine	28–55	-	• Median sternotomy approach • Transventricular chordal disruption via right ventricular apical access	-			100		Severe TR: 100%	
Oh et al. 2024 [20]	Swine	39–48	13	• Percutaneous loop wire cutting technique via inferior vena cava or superior vena cava	4–6 weeks			100		Mild to moderate TR: *n* = 1, Moderate TR: *n* = 1, moderate to severe TR: *n* = 1, Severe TR: *n* = 7, severe to torrential TR: *n* = 1, torrential TR: *n* = 2	
Bai et al. 2010 [21]	Ovine	23–26	7	• Transvenous approach • Chordal disruption using grasping forceps	6 months			100		Moderate TR: *n* = 3 Severe TR: *n* = 4	
*Annular injury or annulus-altering models*											
Kinney et al. 1991 [23]	Mongrel dogs	18–22	14	• Bilateral thoracotomy approach• Transatrial spiral wire leaflet separation	-			100		Mild TR: 1.5 cm spiral, severe TR:2.2 cm spiral	
Xu et al. 2024 [22]	White swine	55–75	Intervention: *n* = 7, control: *n* = 3	• Transvenous approach• Annular stent implantation	3 months			100		Severe: *n* = 7	
Walter et al. 2011 [24]	Yorkshire swine	-	7	• Thoracotomy approach • Endoscopic annular incision (Cardioport)	8 weeks			100		Progressive (from mild to severe): 100%	

### Leaflet-Targeted Injury or Malcoaptation Models

Xie et al. established a chronic canine TR model by surgically resecting one or two TV leaflets along with their chordae tendineae [16]. The severity of TR correlated with the extent of resection, with severe TR appearing immediately and persisting in serial echocardiography. TR was grade IV after anterior leaflet resection and grade IV plus after anterior and posterior leaflet resection. Over follow-up at 3, 6, and 12 months, two-leaflet resection was associated with progressive RA enlargement, tricuspid annular dilation, and RV functional decline by 12 months. Reported follow-up extended to 1 year, during which all animals survived. This model provides durable, graded primary TR with long-term remodeling assessment and high reproducibility.

**Fig. 1:**
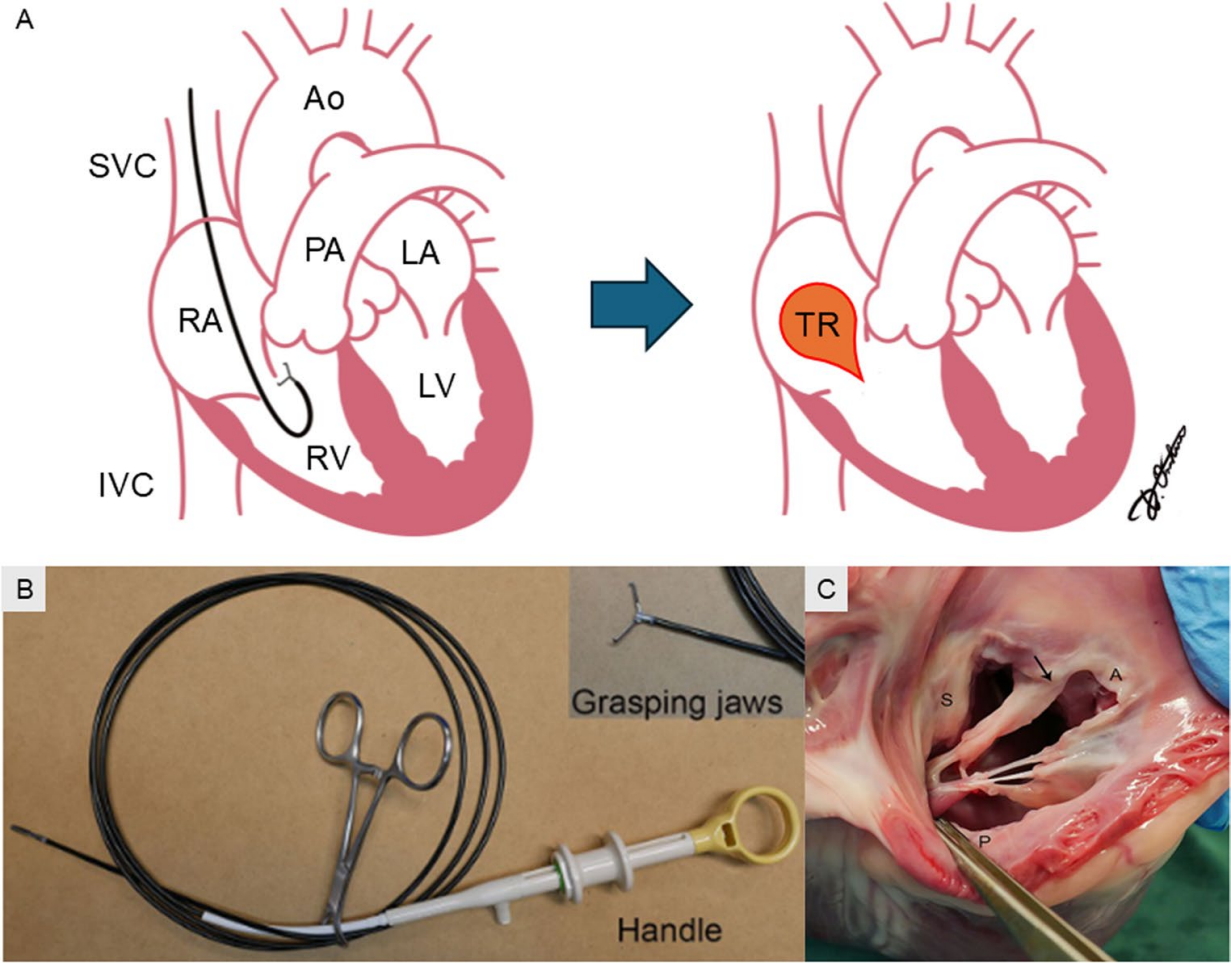
(**A**) Schematic illustration of transcatheter grasping and traction on the tricuspid valve. (**B**) Grasping forceps used in tricuspid regurgitation creation. (**C**) Excised heart showed the anterior leaflet was severely torn (arrow). A, anterior leaflet; S, septal leaflet; P, posterior leaflet; Ao, Aorta; IVC, inferior vena cava; LA, left atrium; LV, left ventricle; PA, pulmonary artery; RA, right atrium; RV, right ventricle; TR, tricuspid regurgitation. These images were adapted/reproduced from Ref [16]

Yan et al. reported a percutaneous swine model in which TR was created by transcatheter grasping and traction on the TV and subvalvular apparatus (Fig. 1A) using grasping forceps (Fig. 1B) advanced through an 8.5-Fr steerable guide [16]. Severe TR was achieved in 90.91% (10/11) of animals, with moderate-to-severe TR in all animals immediately after induction. Transient VT or VF occurred in 63.64% of animals during induction, with conversion to sinus rhythm either spontaneously or by defibrillation, and there were no procedure-related deaths or deaths during 1-month follow-up. Autopsy findings confirmed leaflet tears with occasional chordae tendineae disruption and no papillary muscle injury (Fig. 1C), highlighting a lesion pattern that can vary depending on the tissue engaged during the grasp-and-withdraw maneuver. This percutaneous model reliably generates severe primary TR, although lesion morphology can vary and intraprocedural arrhythmia remains an important limitation.

Heerdt et al. developed an acute canine TR model to investigate the impact of TR on thermodilution cardiac output measurements [17]. TR was induced by immobilizing one or two leaflets using sutures placed through the targeted leaflet and its corresponding papillary muscle and exteriorized through the RV free wall. This approach enabled stepwise augmentation of TR from moderate to severe by immobilizing one versus two leaflets, with TR severity graded using retrograde inferior vena cava (IVC) flow-based criteria. This model is useful for controlled acute hemodynamic studies with stepwise TR augmentation, but chronic survival and remodeling were not evaluated.

### Subvalvular Disruption Models

**Fig. 2:**
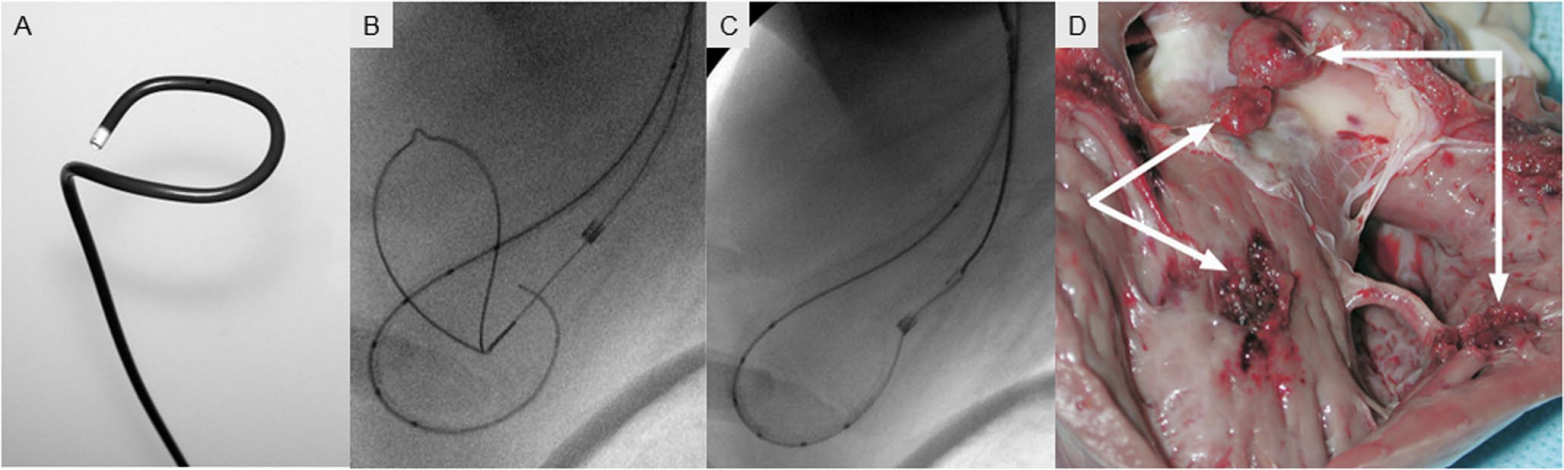
(**A**) The modified left coronary catheter with an additional secondary right-angle curve. (**B**) Fluoroscopic image shows that 0.035-inch stainless steel guide wire through modified left coronary catheter is ready to be snared by a goose-neck snare, forming a wire loop underneath papillary muscle. (**C**) The tip of the guide-wire is snared and partially retrieved to form a closed loop. (**D**) Autopsy image showing avulsion of two papillary muscles (white arrow). These images were adapted/reproduced from Ref [18]

Hoppe et al. established a percutaneous ovine TR model by avulsing tricuspid papillary muscle(s) using bilateral jugular venous access to create a transvenous wire loop (Fig. 2A) [18]. After positioning the loop beneath a targeted papillary muscle (Fig. 2B, C), simultaneous traction was applied to achieve papillary muscle avulsion. Technical success was reported in all cases, but one animal died within minutes after acute TR onset, indicating limited tolerance to abrupt severe regurgitation in some cases. Although regurgitation was reliably induced, TR severity was not quantitatively graded by echocardiography. This model serves as an acute percutaneous proof-of-concept for papillary muscle disruption (Fig. 2D) without thoracotomy, but survival and remodeling assessment were not established.

Miller et al. created an acute porcine TR model by transventricular disruption of the chordal apparatus through RV apical access after sternotomy [19]. A nerve hook introduced through an apical purse-string was advanced to engage the TV supporting structures and then pulled or sectioned to disrupt the chordae, and regurgitation severity was increased by additional chordal cuts. TR could be generated over a range from mild to severe in a stepwise manner, enabling controlled hemodynamic perturbation. This model permits graded acute induction of TR for hemodynamic studies, but it requires an invasive open-chest approach and has not been tested for survival outcomes.

Oh et al. developed a porcine model using a percutaneous loop wire cutting technique via either the IVC or superior vena cava (SVC) route to disrupt the tricuspid subvalvular apparatus and adjacent valve structures [20]. A guidewire–snare system was used to form a wire loop across the chordal apparatus under fluoroscopic guidance, and loop actuation produced structural disruption and regurgitation. Moderate-to-torrential TR was induced in 12 of 13 animals and graded by echocardiography. Animals survived to 4–6 weeks with sustained TR and progressive right-heart enlargement on echocardiography and computed tomography. A distinctive feature of this model was that the access route influenced jet direction, with SVC access tending to produce posterolateral jets and IVC access tending to produce septally directed jets. This model supports short-term survival studies after percutaneous structural disruption, with sustained TR and imaging-based assessment of right-heart remodeling. Practical limitations include imprecise control over the extent of structural injury and the final TR severity, which can vary widely.

Bai et al. reported a percutaneous ovine TR model via femoral venous access using grasping forceps to engage chordae tendineae or leaflet tissue under image guidance [21]. After TR was confirmed with gentle traction, the forceps were forcibly withdrawn to disrupt the targeted structures and induce regurgitation. More than moderate TR was achieved in all animals, including severe TR in a subset, with no procedure-related deaths and no mortality during 6-month follow-up. Injury predominantly involved the anterior leaflet, while the septal leaflet remained intact, a potentially relevant feature given the proximity of the cardiac conduction system to the septal leaflet attachment. Despite persistent TR, overall hemodynamic parameters remained stable over follow-up. This model provides a minimally invasive platform for durable TR induction with longer-term follow-up, although mixed lesion distribution and limited control over the disrupted leaflet or chordal elements remain important limitations.

### Annular Injury or Annulus-Altering Models

**Fig. 3:**
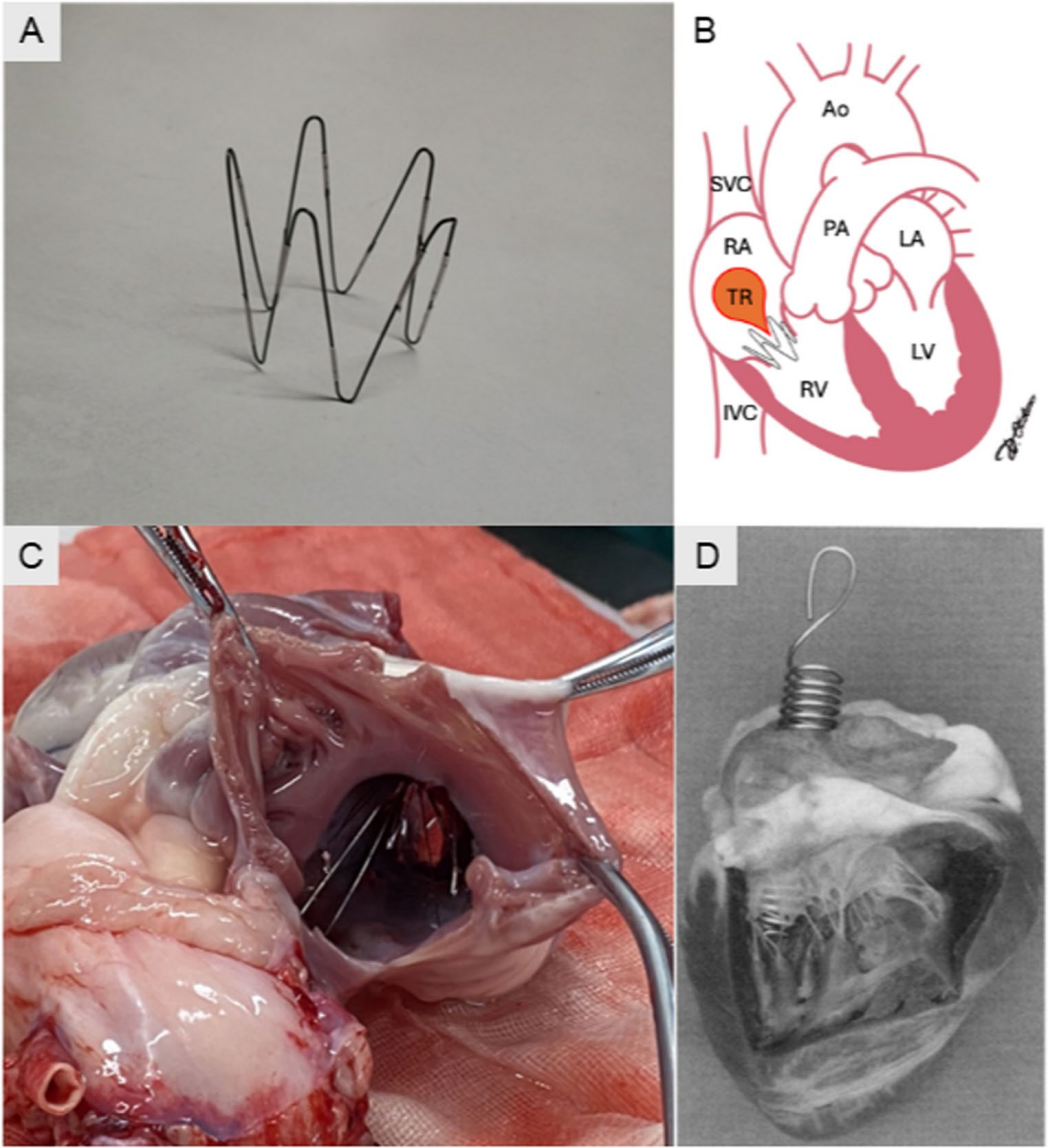
(**A**) A circular self-expanding nitinol stent constructed from a shape-memory alloy wire. (**B**) Schematic illustration of the self-expanding stent–based approach used to create tricuspid regurgitation. (**C**) A right atrial incision demonstrates the stent supporting the tricuspid annulus and partially surrounding the chordae tendineae. These images were adapted/reproduced from Ref [22]. (**D**) An image of the excised heart demonstrates the position of the spiral, which is advanced through the right atrial wall in a corkscrew fashion, across the atrioventricular canal, and into the right ventricle. This image was adapted/reproduced from Ref [23]

Xu et al. created TR in a swine model using a self-expanding stent–based approach [22]. A circular self-expanding stent fabricated from a nickel–titanium shape-memory alloy wire (Fig. 3A) was delivered through the internal jugular vein and deployed under fluoroscopic guidance (Fig. 3B). All animals survived the procedure and follow-up period, and severe TR developed in all swine in the experimental group. During follow-up, TR severity, RA area, tricuspid annular diameter, and indices of RV function progressively worsened. At necropsy, all implanted stents were located at the tricuspid annulus and attached to TV leaflets and chordae tendineae, with partial myocardial embedding in some cases (Fig. 3C). This model provides a percutaneous and reproducible method for generating severe TR with progressive right-heart remodeling, although the permanent intracardiac implant and its interaction with leaflet and chordal structures are important limitations.

Kinney et al. described an acute, reversible canine TR model that uses a spiral wire to prevent complete TV leaflet apposition after surgical exposure (Fig. 3D) [23]. TR severity was titrated by wire diameter, with a 1.5-cm spiral wire producing mild TR and a 2.2-cm spiral wire producing severe TR, and valvular competence could be restored by withdrawing the wire. Key advantages include reversibility and the ability to repeatedly induce graded TR within the same animal without procedural mortality. These features make the model particularly useful for acute hemodynamic studies requiring controlled and repeatable TR induction.

Walter et al. reported a porcine TR model created by producing 3–5-mm incisions in the tricuspid annulus using a custom-made optical port system called Cardioport [24]. The Cardioport was introduced into the RA via thoracotomy, and multiple annular incisions were created under cardioscopic and echocardiographic guidance while avoiding injury to the valve leaflets and subvalvular apparatus. Acute TR was successfully induced in all animals and was well tolerated without procedural mortality or major morbidity. Over an 8-week follow-up period, annular diameter progressively increased from 23.1 ± 1.7 mm preoperatively to 37.3 ± 3.6 mm, and TR progressed from mild to severe in all animals. This model enables progressive annular dilation and chronic TR without direct leaflet or subvalvular injury, but it requires thoracotomy and a specialized Cardioport imaging system.

### Indirect Hemodynamic Models (Secondary/Functional TR)

Indirect models induce TR through altered right-sided loading conditions, myocardial dysfunction, or other mechanisms that impair leaflet coaptation without primary structural injury to the valve. These approaches are useful for studying atrial and ventricular FTR mechanisms and for evaluating interventions in chronic remodeling models, although they often involve greater TR variability and longer time courses (Table 2).

**Table 2:** Large animal models of secondary (functional) tricuspid regurgitation based on indirect intervention models

Author, year [ref#]	Animal species	Body weight (kg)	Animal number	Methods	Duration	Survival rate [%]	TR
*Acute tethering or malcoaptation models*							
Buffington et al. 2004 [25]	Swine	23–26	5	• Median sternotomy approach • Snaring the free-wall leaflets with two sutures	-	100	Not assessed
Onohara et al. 2024 [26]	Yorkshire farm swine	87.8 ± 3.9	5	• Right thoracotomy approach • Chordal encircling snares via right ventricular myocardium	Maximum of 6 hours	100	Severe TR: 100%
*Pressure- and volume-overload models (right-sided loading)*							
Ascione et al. 2026 [27]	Swine		8	PA banding (Umbilical tape)	Acute	100	Median FTR increasing from 1 to 3
Gaweda et al. 2023 [28]	Ovine	62 ± 7	17	PA banding (Umbilical tape)	8 weeks	85 (17/20)	Mild TR: *n* = 3 Moderate TR: *n* = 3 Severe TR: *n* = 11
Malinowski et al. 2018 [30]	Ovine	Group 1: 56 ± 6 Group 2: 50 ± 2	Group 1: *n* = 10 Group 2: *n* = 6	Volume infusion (500 mL crystalloid solution) + PA constriction (PA pneumatic occluder) + Ischemic (PDA occlusion) Group 1. stepwise application of the three Group 2. combined (simultaneous) application	Five minutes for each method	100	Group 2: TR increased from 0.4 ± 0.5 to 2.5 ± 0.8 (*p* < 0.001)
Lin et al. 2024 [1]	Swine	80.5 ± 7.5	12	Ablation (endocardium, > 80% of RA and < 30% of LA) + atrial septostomy (left-to-right shunt, > 1 cm) + right heart volume overload (daily 2,000 mL of saline)	1 month	100	Mild TR: *n* = 3 Moderate TR: *n* = 8 Severe TR: *n* = 1
*Tachycardia-induced cardiomyopathy models (rapid pacing)*							
Malinowski et al. 2017 [31]	Ovine	61 ± 4	6	LV pacing [220–240 bpm, from 5 ± 1 days after Sonomicrometry implant until LVEF became < 30% and there was presence of TR (+ 2 or greater)]	14 ± 5 days	85.7 (6/7) (3/10 animals died before starting pacing)	Moderate TR: *n* = 3 Moderate to Severe TR: *n* = 2 Severe TR: *n* = 1
Jazwiec et al. 2021 [32]	Ovine	64 ± 5	9	LV pacing [200–240 bpm, from 4–5 days after Sonomicrometry implant until LVEF became < 30% and there was presence of TR (+ 2 or greater)]	15.5 ± 3.5 days	81.8 (9/11) (9/20 animals were excluded due to pacing failure, etc.)	Median FTR increased from 0 to + 3 (*p* = 0.004)

### Acute Tethering or Malcoaptation Models

Buffington et al. developed an acute swine model of tricuspid malcoaptation by placing two 3 − 0 Prolene sutures through the RV free wall to snare the free-wall leaflets of the TV [25]. Controlled traction on these sutures impaired leaflet coaptation and produced significant regurgitation without compromising global cardiac motion. The sutures were secured to an external frame using rubber bands, allowing sustained and adjustable tension throughout the experiment. In this study, the presence of significant TR was demonstrated by a reduction in calculated forward stroke volume; however, TR severity was not quantitatively assessed. This animal model is useful for controlled acute studies of malcoaptation-related TR physiology, although it does not reproduce the progressive right-heart remodeling characteristic of FTR.

**Fig. 4:**
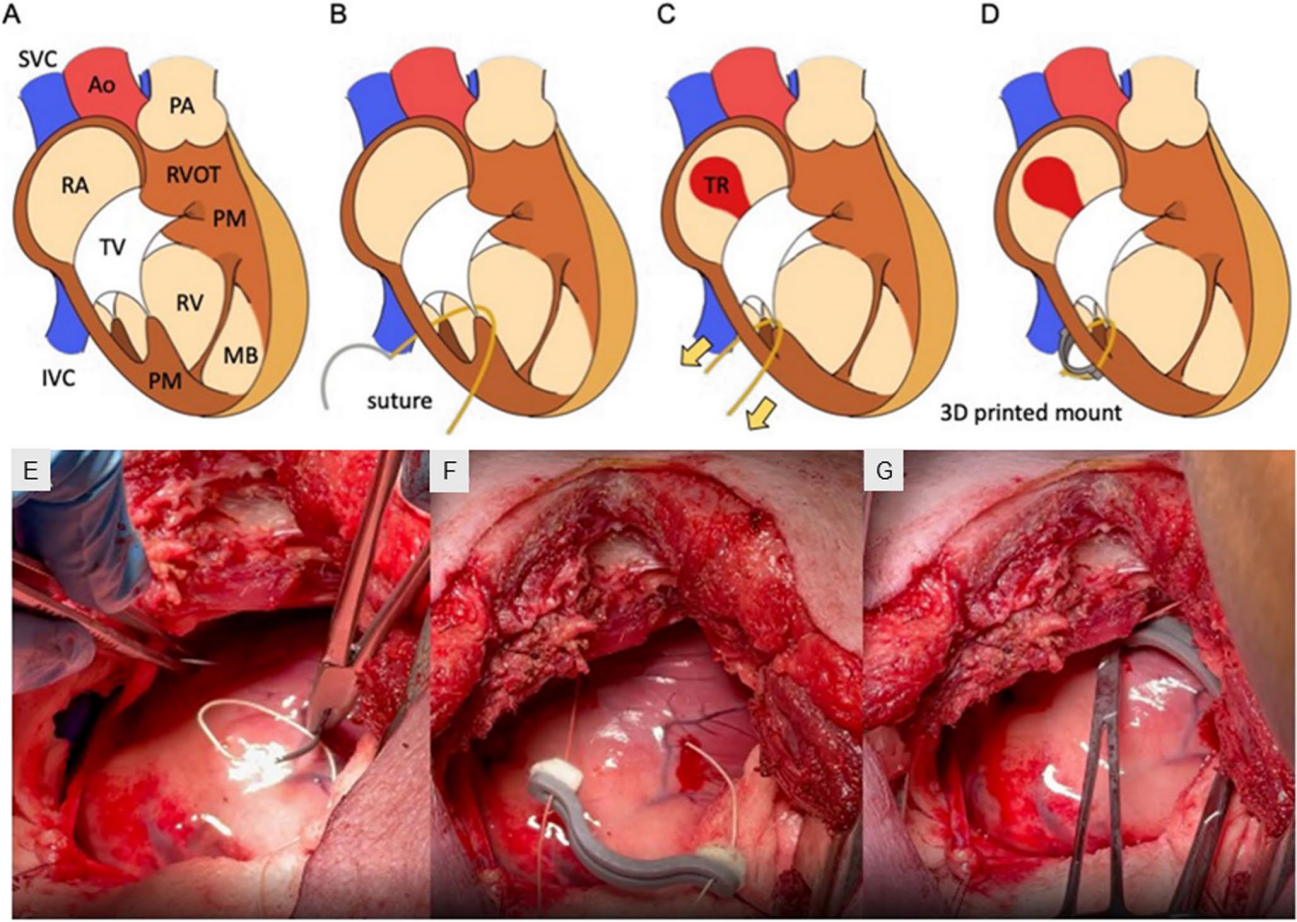
(**A**) A schematic illustration of the right heart anatomy. MB, moderator band; PM, papillary muscle; RVOT, right ventricular outflow tract; TV, tricuspid valve. (**B**) A chordal-encircling snare was advanced through the right ventricular free wall to encircle the tricuspid chordae tendineae. (**C**) The snare was exteriorized through two papillary muscles located on the RV free wall. (**D**) The free ends of the suture loop are tied onto a 3D-printed mount. (**E**) Intraoperative image shows a suture is inserted through the RV free wall. (**F**) The free ends of the suture are passed through a 3D-printed mount. (**G**) The free ends of the suture loop are tied onto this mount. These images were adapted/reproduced from Ref [26]

Onohara et al. reported a swine model of FTR created by leaflet tethering using image-guided chordal encircling snares placed through the RV myocardium via right thoracotomy and secured to a custom 3D-printed epicardial mount (Fig. 4) [26]. Severe FTR was successfully induced in all animals without procedural complications, arrhythmias, or mortality, and all animals remained hemodynamically stable for up to 6 h. This technique produced leaflet tethering with loss of coaptation between the septal leaflet and the other leaflets, increased tricuspid annular diameter and tenting area, and enlargement of RV dimensions. This model enables reproducible acute induction of severe FTR with controlled leaflet tethering and associated right-heart geometric changes, although chronic remodeling was not evaluated.

### Pressure- and Volume-Overload Models (Right-Sided Loading)

Ascione et al. demonstrated that partial PA banding can acutely induce significant FTR in swine [27]. Partial banding increased RV pressure and caused dilation of the RV and tricuspid annulus, particularly the septo-lateral diameter. At least moderate-to-severe TR was achieved in all animals, median TR grade increased from 1 to 3, and two swine developed greater-than-severe TR. All animals survived the procedure, and the induced changes remained stable for up to one hour. This approach enables simple acute induction of significant FTR in swine, but its relevance to chronic remodeling remains unknown.

**Fig. 5:**
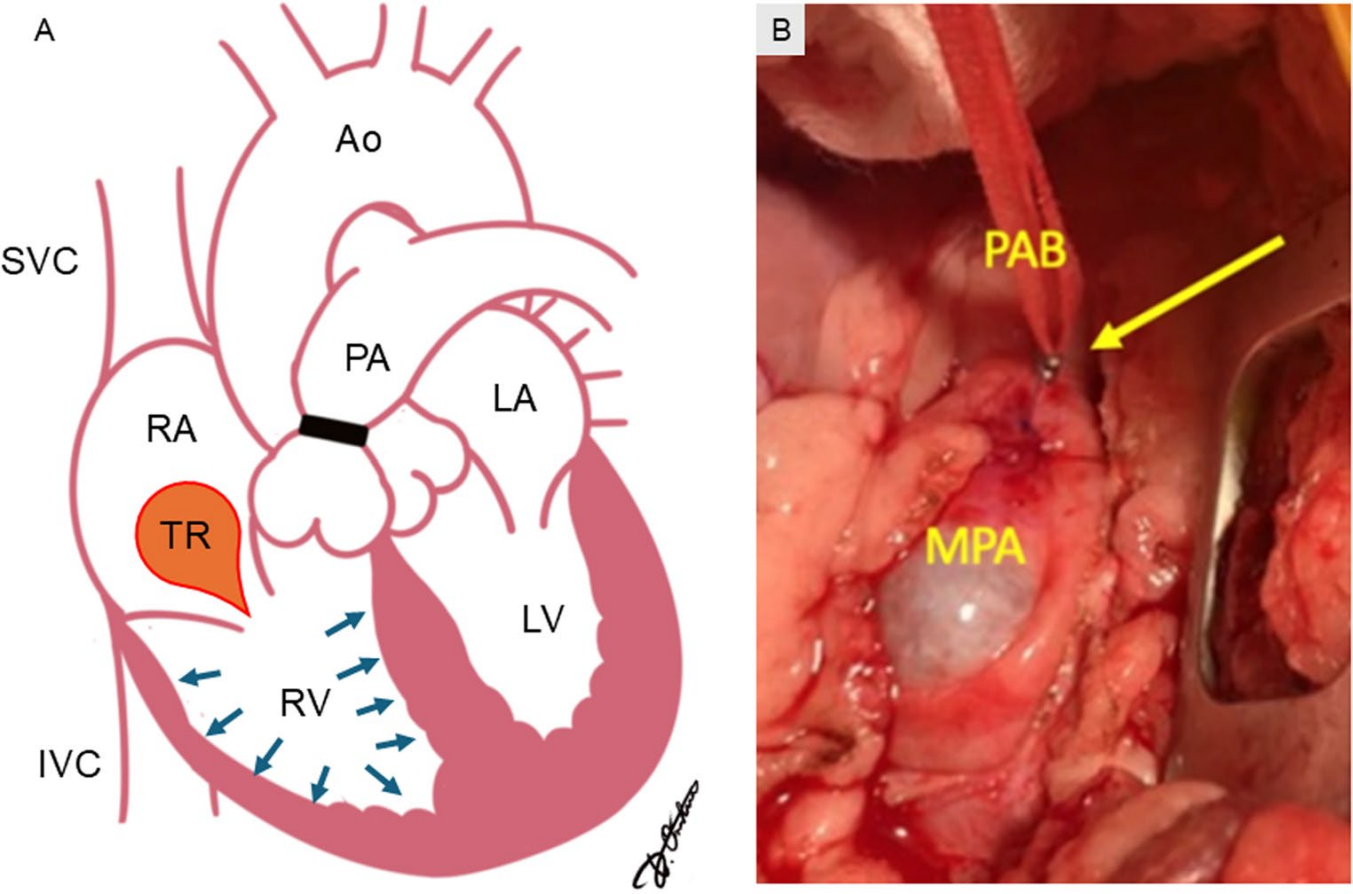
(**A**) Schematic illustration of FTR induced by pulmonary artery banding. (**B**) Intraoperative image showing pulmonary artery banding performed with an umbilical tape (yellow arrow) passed around the main pulmonary artery and secured with a clip. MPA, main pulmonary artery; PAB, pulmonary artery band. Panel B was adapted from Ref [28]

Gaweda et al. developed a chronic ovine model using PA banding as the sole pressure-overload method to induce RV failure and FTR (Fig. 5) [28]. Adult male sheep underwent PA banding and were followed for 8 weeks with surveillance echocardiography. Survival at 8 weeks was 85% (17/20). Among survivors, 3 animals developed mild TR, 3 developed moderate TR, and 11 developed severe TR. This chronic ovine model is characterized by sustained right-heart remodeling and a high rate of moderate or severe FTR, although mortality during follow-up remains an important limitation.

Malinowski et al. reported an acute ovine model in which RV failure and greater than moderate FTR were induced by combining three interventions: rapid volume loading with 500 mL crystalloid over 2 min, pressure overload by PA constriction targeting at least a 50% increase in RV pressure for 5 min, and ischemic induction by posterior descending artery occlusion for 5 min [29, 30]. When applied individually, none of these interventions produced RV failure or clinically significant TR; when combined, however, they resulted in RV failure with greater than moderate FTR. This acute combined-intervention model reproduces RV failure with FTR, but it requires multiple simultaneous interventions in an open-chest setting.

**Fig. 6:**
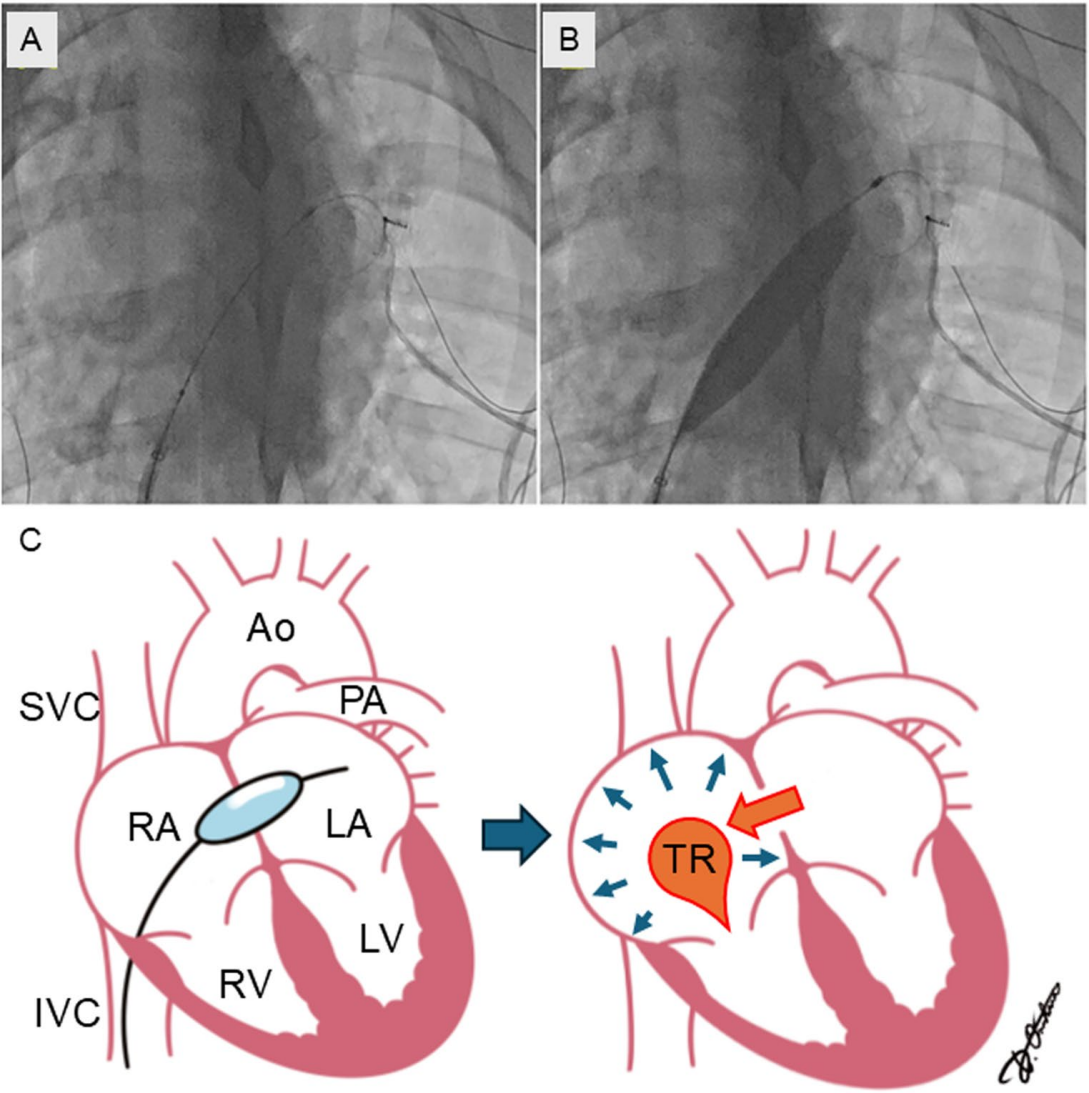
(**A**, **B**) Interatrial fistulization created by balloon catheter dilation to induce right heart volume overload. The images were adapted/reproduced from Ref [1]. (**C**) Schematic illustration of interatrial fistulization. Ao, aorta; IVC, inferior vena cava; LA, left atrium; LV, left ventricle; PA, pulmonary artery; RA, right atrium; RV, right ventricle; TR, tricuspid regurgitation

Lin et al. developed a swine model of FTR using an interatrial left-to-right shunt (Fig. 6A-C), radiofrequency ablation of the left and right atrial endocardium, and daily administration of 2,000 mL saline for 1 month [1]. At 1 month after the intervention, all animals had TR on echocardiography, including mild TR in 3, moderate TR in 8, and severe TR in 1. There were also significant increases in TV annular size and tenting height, as well as enlargement of the RA and RV, while there was no significant change in left-sided heart parameters. This swine model is suited to chronic studies of FTR with annular and right-heart remodeling without direct structural valve injury, but the need for three separate interventions makes it more complex to implement than single-mechanism loading models.

### Tachycardia-Induced Cardiomyopathy Models (Rapid Pacing)

Malinowski et al. developed an ovine model of FTR by inducing tachycardia-induced cardiomyopathy with high-rate pacing at 220–240 bpm using a monopolar pacing lead sutured to the anterior left ventricular (LV) wall [31]. Pacing continued until LVEF decreased to less than 30% and significant TR was observed on echocardiography. Of 10 animals, 3 died during early recovery and 1 died during the pacing phase, whereas the remaining 6 animals completed a mean pacing period of 14 ± 5 days. All surviving animals developed at least moderate TR, accompanied by biventricular dysfunction, tricuspid annular dilation, RV enlargement, and increased mitral regurgitation. This model is particularly useful for studying FTR in the setting of end-stage biventricular failure rather than isolated right-heart disease.

Using a similar ovine tachycardia-induced cardiomyopathy model, Jazwiec et al. later investigated the effect of variable tricuspid annular reduction on FTR and RV dynamics [32]. Animals were paced at 200–240 bpm for 15.5 ± 3.5 days until LVEF decreased to less than 30% and at least moderate FTR developed. Among 20 study animals, 9 completed the protocol, 2 died during pacing, and 9 were excluded because of pacemaker failure, crystal damage, insufficient HF induction, or inability to wean from cardiopulmonary bypass. Progressive annular reduction reduced TR, but aggressive reductions were associated with deterioration of RV function and strain. A notable limitation of this experimental platform is the high attrition rate related to the complexity of the pacing and terminal study protocol.

## Model Selection, Translational Implication, and Future Directions

This review highlights that currently available animal models of TR differ substantially in how closely they reproduce the clinical phenotypes of the disease. Historically, many experimental TR models have relied on direct manipulation of the tricuspid leaflets or subvalvular apparatus, as these approaches allow immediate and technically controlled induction of regurgitation. However, because most clinical TR is functional [1, 2], leaflet-preserving models that reproduce remodeling-driven TR are particularly important for translational research. Selected volume-overload strategies can reflect clinical settings characterized by fluid retention and chamber dilation [13]. In addition, pacing-based approaches and selected atrial remodeling strategies may provide alternative means of inducing remodeling-associated TR, although the resulting phenotype depends on the specific model used. Thus, model selection should be guided not only by the ability to induce TR, but also by how well a given model reproduces the specific pathophysiological phenotype required for the intended experimental purpose.

FTR should not be regarded as a single entity. At least two representative phenotypes are recognized. PH, PA banding, and other RV pressure-overload models generally correspond more closely to ventricular FTR, as they primarily induce RV remodeling and leaflet tethering. In contrast, atrial arrhythmia-based models and selected volume-overload models may reproduce some features of atrial FTR. This distinction is important when selecting animal models. In addition, in patients with severe TR without PH, RV dysfunction has been identified as the strongest predictor of mortality [33], further underscoring the importance of RV phenotype in model selection.

Across currently available FTR models, there is a tradeoff between reproducibility and translational realism. PA banding is one of the most reproducible methods and offers acceptable survival, but it primarily reflects pressure-overload driven ventricular remodeling. Combined pressure- or volume-overload strategies with ischemic injury can induce substantial TR, but these approaches are more complex, and their mechanistic attribution is less straightforward. Pacing models may be useful for device testing in global HF, but they are less suitable for isolated right-sided FTR investigations.

Additionally, species-specific characteristics should be considered when selecting large animal TR models. Canine and ovine models have provided valuable information on chronic remodeling and long-term outcomes, but their use may be limited by cost, ethical considerations, and animal availability. Swine are commonly used in cardiovascular research and are attractive for translational studies because of their availability, cardiovascular size, and compatibility with surgical and catheter-based procedures. However, adapting canine or ovine TR-induction techniques to swine requires attention to rapid somatic growth and procedure-related arrhythmias. These challenges can be mitigated by using miniature pigs or age- and weight-matched cohorts for chronic studies and by maintaining continuous rhythm monitoring with readiness for defibrillation and antiarrhythmic therapy.

PH-associated TR typically develops over time through progressive RV remodeling. In this setting, TR appears to result mainly from RV anatomical and functional alterations, including annular dilation and leaflet tethering [34]. PH-based models are therefore useful for studying FTR driven by RV pressure overload and remodeling, often in the absence of primary LV systolic dysfunction. However, these models should be interpreted as representing one pathophysiological pathway within the broader spectrum of FTR.

In patients with FTR, the tricuspid annulus becomes more planar, more circular, and dilated predominantly in the septal-to-lateral direction, compared with the nonplanar, saddle-shaped annulus seen in healthy subjects [35–37]. From this perspective, future model development should focus on leaflet-preserving platforms that reproduce the geometric features of ventricular FTR without disrupting the tricuspid apparatus. Such models may be particularly valuable for translational investigation and preclinical intervention testing. The FTR model reported by Onohara et al. [26] is of interest in this regard because it deforms the RV and TV apparatus together while preserving the native leaflets and subvalvular structure, although chronic remodeling was not evaluated.

When discussing experimental models of TR, it is essential to recognize the heterogeneity of TR etiologies and phenotypes. Investigators should first define the clinical phenotype and pathophysiological sequence they aim to reproduce, as well as the intended use of the model, and then select the modeling strategy best suited to that purpose.

## Conclusion

Animal models are indispensable for elucidating the pathophysiology of TR and for the preclinical evaluation of therapeutic interventions. Existing models have provided important insights into TR induction and mechanisms, but no single model captures the full spectrum of clinically relevant disease. Most clinical TR is functional, underscoring the need for leaflet-preserving models that better reproduce ventricular and atrial FTR phenotypes, particularly in the setting of RV dysfunction or PH.

## Data Availability

No new data were generated or analyzed in this review. All data discussed are from published studies cited in the manuscript.

## Abbreviations

Ao: aorta
FTR: functional tricuspid regurgitation
IVC: inferior vena cava
HF: heart failure
LA: left atrium
LV: left ventricle
LVEF: left ventricular ejection fraction
PA: pulmonary artery
PDA: posterior descending artery
PH: pulmonary hypertension
RA: right atrium
RV: right ventricle
RVOT: right ventricular outflow tract
SVC: superior vena cava
TR: tricuspid regurgitation
TV: tricuspid valve
VF: ventricular fibrillation
VT: ventricular tachycardia
